# N‐terminal pro‐brain natriuretic peptide and short‐term mortality in acute aortic dissection: A meta‐analysis

**DOI:** 10.1002/clc.23436

**Published:** 2020-07-31

**Authors:** Mislav Vrsalovic, Ana Vrsalovic Presecki, Victor Aboyans

**Affiliations:** ^1^ University of Zagreb School of Medicine Zagreb Croatia; ^2^ Department of Cardiology Sestre Milosrdnice University Hospital Center Zagreb Croatia; ^3^ Faculty of Chemical Engineering and Technology University of Zagreb Zagreb Croatia; ^4^ Department of Cardiology Dupuytren University Hospital Limoges France; ^5^ INSERM 1094 Limoges University Limoges France

**Keywords:** aortic dissection, brain natriuretic peptide, meta‐analysis, mortality

## Abstract

**Background:**

Acute aortic dissection (AAD) is a life‐threatening medical emergency that requires immediate diagnosis and rapid treatment. There is a paucity of data on the role of biomarkers in risk stratification of patients with AAD.

**Hypothesis:**

N‐terminal pro‐brain natriuretic peptide (NT‐proBNP) is associated with short‐term mortality in AAD patients.

**Methods:**

We systematically searched Medline and Scopus to identify all observational cohort studies published before January 2020 that compared outcome (short‐term mortality) in patients with AAD with high vs low levels of baseline NT‐proBNP combining terms “brain natriuretic peptide” and “aortic dissection.” A meta‐analysis was conducted using the generic inverse variance method. Heterogeneity between studies was investigated using the Cochrane's Q test and *I* ^*2*^ statistic.

**Results:**

Four studies were included in final analysis including a total of 950 patients, and 105 (11%) patients died. Baseline NT‐proBNP concentrations were significantly higher in nonsurvivors (median 2240 pg/mL, range 1678‐16 347 pg/mL) when compared to survivors (665 pg/mL, 328‐1252 pg/mL). Elevated NT‐proBNP values were significantly associated with an increased risk of short‐term mortality (odds ratio 4.13, 95% CI [confidence interval] 2.33‐7.33), with low heterogeneity (*I* ^*2*^ = 8.77%, Cochran Q = 2.19, *P* = .33), and no publication bias. The pooled standardized mean difference between groups was 1.28 (95% CI 0.99‐1.56), with low heterogeneity (*I* ^*2*^ = 38.73%, Cochran Q = 3.26, *P* = .19).

**Conclusion:**

Elevated NT‐proBNP levels on admission are associated with an increased risk of short‐term mortality in AAD.

## 1 INTRODUCTION

Acute aortic dissection (AAD) is a life‐threatening medical emergency that requires immediate diagnosis and treatment.^1^ Circulating biomarkers are attractive tools in diagnostic decision making and risk stratification in AAD. Currently, there is a paucity of data on the role of biomarkers in risk stratification of patients with AAD. A recent meta‐analysis found that elevated cardiac troponins were associated with an increased risk of in‐hospital mortality in patients with AAD.^2^ Although N‐terminal pro‐brain natriuretic peptide (NT‐proBNP) is widely used in routine cardiology clinical practice its prognostic role in AAD has not yet been systematically investigated.^3^ Therefore, we performed a systematic review and meta‐analysis to assess whether NT‐proBNP on patient's admission was associated with an increased risk of death in patients with AAD.

## 2 METHODS

### 2.1 Search strategy, study inclusion, and outcomes

This meta‐analysis was performed in accordance with the PRISMA statement.^4^ We systematically searched Medline and Scopus to identify all observational cohort studies published before January 2020 that compared outcome (short‐term mortality) in patients with AAD (presented within 14 days of symptom onset) with high vs low levels of baseline NT‐proBNP combining terms “brain natriuretic peptide” and “aortic dissection.” Admission NT‐proBNP levels were measured by Elecsys 2010 Roche Diagnostics assay. The cutoff values used in the studies varied between 312 and 647 pg/mL.

### 2.2 Data extraction and quality assessment

Study selection and data extraction were conducted independently by two investigators (M. V. and A. V. P.). Any disagreements or differences in the data extraction between the two authors were harmonized by consensus after rechecking the source data. Study quality was assessed using the validated Newcastle‐Ottawa Scale for assessment of nonrandomized and observational studies, and studies were evaluated based on subject selection, comparability of study groups and assessment of the outcome.^5^ Completed database contained the following data: name of the first author, year of publication, country of origin, study design, total number of pts in each study, age, gender, the proportion of patients with hypertension, mortality, type of AAD, NT‐proBNP cutoff values, and Newcastle‐Ottawa score.

### 2.3 Statistical analysis

A meta‐analysis was conducted using the generic inverse variance method and pooled odds ratio (OR) was reported with 95% confidence interval (CI). Pooled standardized mean difference (SMD) and 95% CI were calculated to assess association between admission NT‐proBNP levels and outcome. Heterogeneity between studies was investigated using the Cochrane's Q test and *I* ^2^ statistic. Statistically significant heterogeneity was considered present at *P* < .10 and *I* ^*2*^ > 50%. Publication bias was assessed graphically using a funnel plot. Analyses were conducted using statistical software MedCalc Version 19.1.5.

## 3 RESULTS

### 3.1 Selected studies and baseline characteristics

**FIGURE 1:**
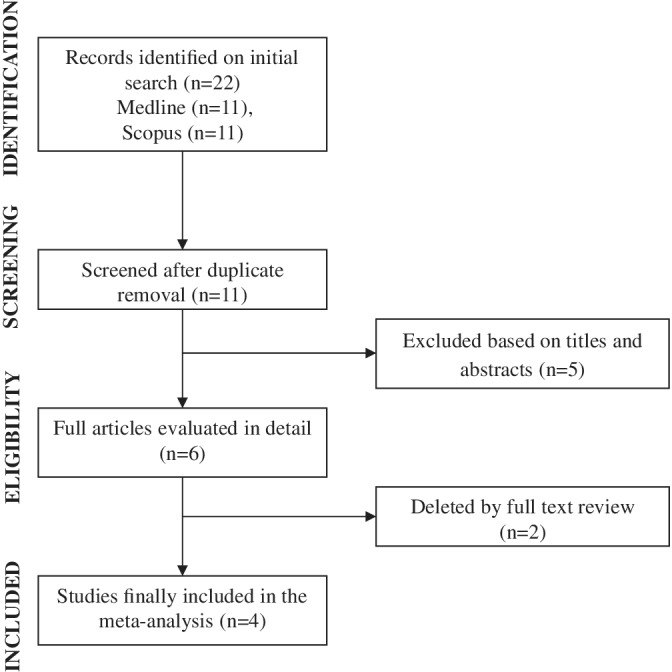
Study flow diagram for meta‐analysis of N‐terminal pro‐brain natriuretic peptide and acute aortic dissection short‐term mortality

Overall, 22 documents were initially identified based on our search criteria. After duplicate removal a total of 11 citations were obtained from electronic search (Figure 1). Based on the title and abstract analysis and review of potentially relevant studies, four studies were included in final analysis,^6^, ^7^, ^8^, ^9^ including a total of 950 patients, of whom 247 (26%) had type A AAD (Table 1). Two studies were retrospective,^7^, ^9^ and two were prospective in nature.^6^, ^8^ In the study of Sodeck et al,^6^ all the patients with type A AAD underwent surgery, and in the study of Zhang et al^9^ 33% received surgery but treatment strategy was not associated with in‐hospital mortality (*P* = .63). The study of Wen et al^8^ included patients with both type A and type B AAD, and surgical treatment was not a predictor of in‐hospital mortality (*P* = .46). The study of Luo et al^7^ included patients with type B AAD. Thoracic endovascular aortic repair was performed in patients with complicated type B AAD (68%), who presented with recurrent or refractory pain, uncontrolled hypertension despite medical treatment, rapid aortic expansion, mal‐perfusion of the viscera or limbs, signs of rupture or hypotension/shock. Median age of the population was 57 years (range 50‐61 years), 75% were males (range 60‐84%), 84% had a history of hypertension, and 105 (11%) patients died. Using the Newcastle‐Ottawa scoring system, for included studies median score was 8 (range 7‐9).

**TABLE 1:** Characteristics of studies included in meta‐analysis

Author, year	Country	Patients (n)	Age (years)	Male (%)	HTN (%)	Study design	Mortality (%)	Dissection type	NT‐proBNP cutoff (pg/ml)	NOS (0–9)
Sodeck, 2008^6^	Austria	104	61	65	NR	P	22	A	647	9
Zhang, 2016^9^	China	67	57	60	78	R	39	A	590	7
Wen, 2019^8^	China	122	50	84	79	P	24	A/B	570	9
Luo, 2019^7^	China	657	57	84	85	R	4	B	312	7

Abbreviations: HTN, hypertension; NOS, Newcastle‐Ottawa Scale; NR, not reported; NT‐proBNP, N‐terminal pro‐B‐type natriuretic peptide; P, prospective; R, retrospective.

### 3.2 Quantitative data synthesis

**FIGURE 2:**
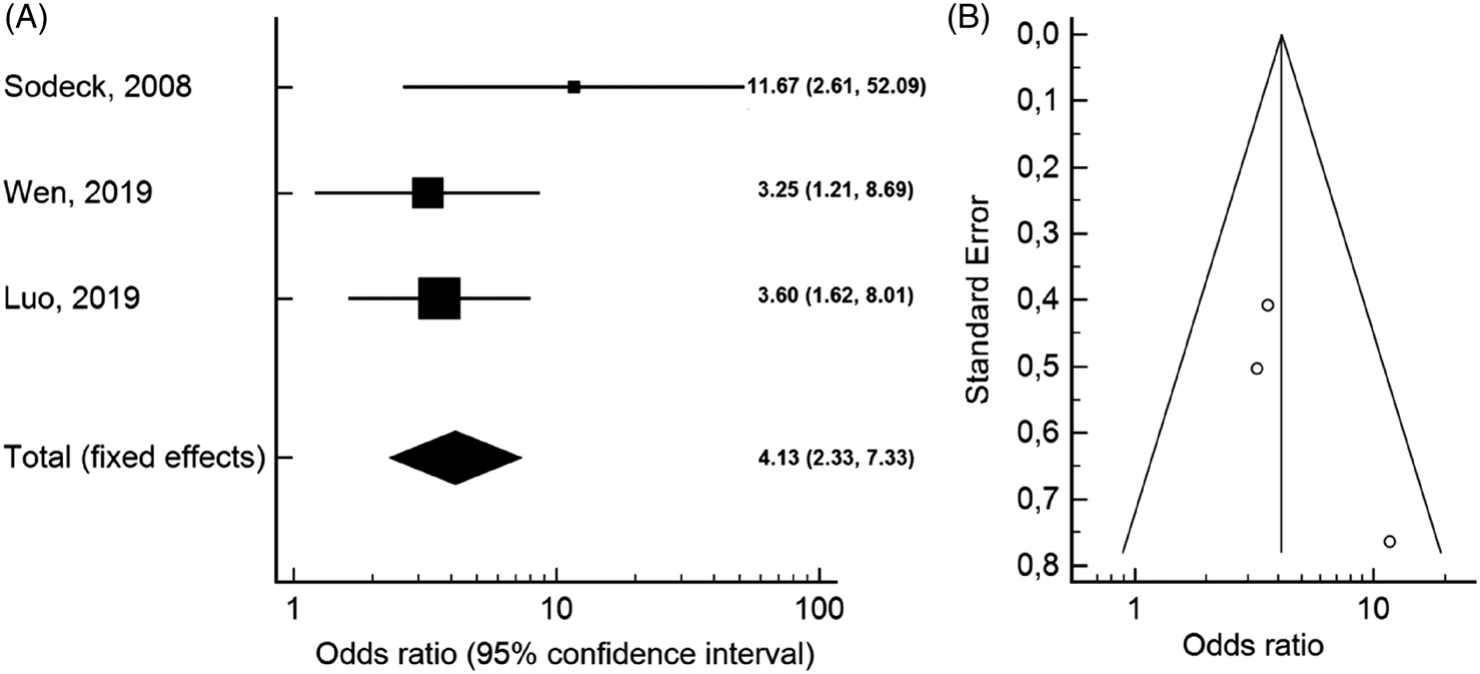
A, Forest plot of odds ratios (ORs) for baseline N‐terminal pro‐brain natriuretic peptide (NT‐proBNP) to predict short‐term mortality in acute aortic dissection (AAD). B, Funnel plot of ORs showing no publication bias. The meta‐analysis was conducted using the generic inverse variance method, and pooled OR was reported with 95% confidence interval. There was no significant heterogeneity observed across studies (*I* ^*2*^ = 8.77%, *P* = .33)

**FIGURE 3:**
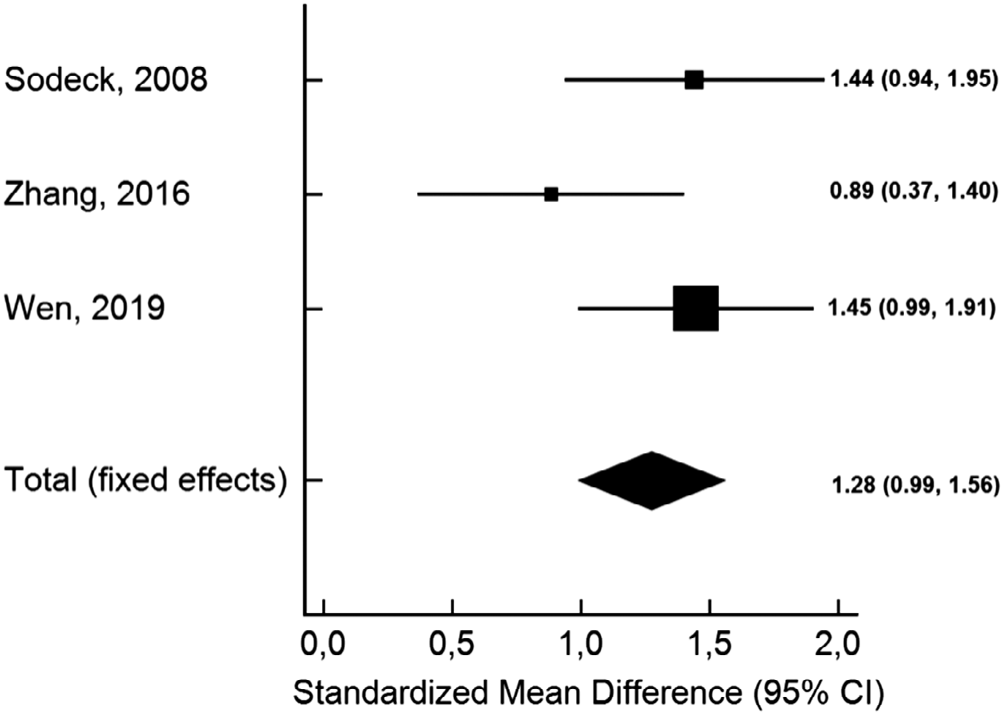
Forest plot of standardized mean differences (SMD) for baseline N‐terminal pro‐brain natriuretic peptide levels and short‐term mortality in acute aortic dissection. The pooled SMD was reported with 95% confidence interval (CI). There was no significant heterogeneity observed across studies (*I* ^*2*^ = 38.73%, *P* = .19)

Baseline NT‐proBNP concentrations were significantly higher in nonsurvivors (median 2240 pg/mL, range 1678‐16 347 pg/mL) when compared to survivors (665 pg/mL, 328‐1252 pg/mL). In the pooled analysis, we found a significant association between elevated NT‐proBNP values and mortality (OR 4.13, 95% CI 2.33‐7.33) (Figure 2A), with low heterogeneity (*I* ^*2*^ = 8.77%, Cochran Q = 2.19, *P* = .33), and no publication bias (Figure 2B). We performed an additional subanalysis of two prospective studies that reported adjusted odds ratios and which included mostly patients with type A AAD (80%).^6^, ^8^ This analysis confirmed the previous results without significant heterogeneity (OR 4.78, 95% CI 2.10‐10.88; Cochran Q = 1.96, *P* = .16). The pooled SMD between groups was 1.28 (95% CI 0.99‐1.56), with low heterogeneity between studies (*I* ^*2*^ = 38.73%, Cochran Q = 3.26, *P* = .19) (Figure 3). Moreover, the pooled SMD of two studies that included only patients with type A AAD confirmed the results (SMD 1.17, 95% CI 0.81‐1.53; Cochran Q = 2.35, *P* = .13).^6^, ^9^

## 4 DISCUSSION

The prognostic role of NT‐proBNP, a well‐established biomarker in everyday cardiology practice, was not comprehensively studied in AAD. According to our meta‐analysis elevated NT‐proBNP on admission is associated with an increased risk of short‐term mortality in AAD.

More than a decade ago, Sbarouni and coworkers reported significantly higher NT‐pro‐BNP values in patients with AAD, compared to healthy subjects, but the underlying mechanisms and its prognostic role remained unclear.^10^

The NT‐proBNP levels are increased in patients with kidney dysfunction due to a decrease in renal clearance, and renal impairment was associated with increased in‐hospital mortality in AAD.^11^, ^12^

The NT‐proBNP levels rise in response to increased ventricular wall stress caused by refractory hypertension (also augmented with recurrent intense pain) and uncontrolled hypertension was associated with adverse in‐hospital outcomes in patients with aortic dissections.^13^ The majority of patients included in our meta‐analysis (84%) had a history of hypertension. Of note, hypertension is both a risk factor and a trigger for AAD. Therefore, it is plausible that most of patients with AAD have long‐standing, resistant, and inadequately controlled hypertension with consequently altered left ventricular (LV) morphology, that is, LV hypertrophy and myocardial strain.^14^ It is well‐known fact that elevated NT‐proBNP values primarily reflect myocardial stretch.^14^ Thus it is possible that LV hypertrophy induces an increase in NT‐proBNP which might be associated with poor prognosis in aortic dissection patients. In addition, from a mechanistic perspective, the NT‐proBNP rise in type A AAD could be related to acute aortic regurgitation due to the volume overload and consequent heart failure.

A significant increase in C‐reactive protein and proinflammatory cytokines was found in aortic dissection patients, and persistent inflammation corresponds to unfavorable outcomes.^15^, ^16^ It is of importance since brain natriuretic peptides are upregulated at the transcriptional and translational levels by proinflammatory cytokines in cardiac myocytes.^17^ Therefore, elevated NT‐proBNP values may serve as an indicator of systemic inflammation with poor clinical outcomes.

So, increased level of NT‐proBNP is a marker of: (a) high‐pressure load from refractory hypertension (also increased by pain) and strain on the left ventricle, (b) heart failure from severe aortic regurgitation in type A AAD, (c) renal dysfunction due to its decreased excretion from the kidney, and (c) inflammation, which altogether may contribute to poor prognosis in aortic dissection patients.^6^, ^7^

The study of Wen et al included patients with both type A and type B AAD, and after multivariable adjustment NT‐proBNP demonstrated its independent prognostic role for in‐hospital mortality.^8^ However, the clear information is missing in the literature about the prognostic value of NT‐proBNP in type A vs type B AAD. Further prospective studies are needed to evaluate the prognostic role of brain natriuretic peptides in the whole spectrum of patients with different presentations of acute aortic syndromes.

Of note, the populations in the included studies were quite homogeneous regarding age, gender, and the percentage of hypertensives, what is of importance regarding the interpretation of NT‐proBNP concentrations. The aortic dissection was most commonly reported in sixth decade of life and predominantly in hypertensive men, in line with the data reported in the International Registry of Acute Aortic Dissection (IRAD).^1^

## 5 STUDY LIMITATIONS

We acknowledge the relatively small number of studies, which were included in our meta‐analysis. However, the fact that an aortic dissection is a rare but life‐threatening cardiovascular disorder and in total 950 patients were included in the analysis, with low heterogeneity and no publication bias between studies, makes our results valuable in everyday clinical setting.

This was a meta‐analysis of observational studies, which is dependent on the methodology of the individual studies that were included. Recently the European Society of Cardiology has lowered their recommended natriuretic peptide thresholds from 400 pg/mL for NT‐proBNP to 125 pg/mL, due to concern that previously recommended thresholds were too high.^18^ Of note, the populations in the studies included in our meta‐analysis were quite homogeneous regarding age, gender, and the percentage of hypertensives what is of importance regarding the interpretation of NT‐proBNP concentrations. However, increased NT‐proBNP levels are associated with aging and comorbidities, and hypertensives have 5 to 6 times higher values comparing to normotensives.^14^ The population included in our meta‐analysis mostly comprised of men (75%) in the sixth decade of life and majority of them had a history of hypertension (84%), what is in line with the data reported in the IRAD. Therefore it is not surprising that in our study median admission NT‐proBNP cutoff was 580 pg/mL.

The four papers included in our study addressed only the prognostic aspect of NT‐proBNP, as they only included patients with diagnosed AAD, and we are not aware of patients with suspicion of AAD. Hence, our article is only focused on prognosis, and the integration of NT‐proBNP in the diagnostic strategy requires future research.

## 6 CONCLUSION

The results of this systematic review and meta‐analysis clearly showed the prognostic value of admission NT‐proBNP elevation for short‐term mortality in patients with AAD. Consequently, NT‐proBNP may serve as an additional risk stratification tool in daily clinical setting in patients with AAD.

## CONFLICT OF INTERESTS

The authors declare no conflict of interest. This research did not receive any specific grant from funding agencies in the public, commercial, or not‐for‐profit sectors.
